# KIF4A in disease pathogenesis and therapeutics: from molecular mechanisms to clinical translation

**DOI:** 10.1186/s13062-025-00712-0

**Published:** 2025-12-08

**Authors:** Yi Liu, Yunhua Li, Chunrong Tang, Honghua Wen, Jingxian Tang, Gangwen Chen, Yongkang Wu

**Affiliations:** 1 Department of Radiology, West China Hospital Sichuan University Jintang Hospital, Jintang First People’s Hospital, Chengdu, Sichuan 610400 China; 2 Department of Laboratory Medicine, West China Hospital, Sichuan University, Chengdu, China

**Keywords:** KIF4A, Tumour microenvironment remodelling, Context-dependency, Precision therapy

## Abstract

Kinesin family member 4 A (KIF4A) is a multifunctional motor protein essential for chromosome condensation, spindle dynamics, and cytokinesis. Beyond its classical mitotic functions, emerging evidence positions KIF4A as a central regulator of tumorigenesis, therapy resistance, metabolic reprogramming, and immune modulation across diverse cancer types. However, no comprehensive review has integrated its molecular mechanisms with its roles in both oncological and non-oncological diseases, nor clarified its context-dependent behavior, including paradoxical tumor-suppressive effects in cervical cancer. In this review, we synthesize current advances spanning structural biology, transcriptional and post-translational regulation, and pathway-level interactions involving PI3K/AKT, TGF-β/Smad, Hippo-YAP, metabolic remodeling, and DNA damage response networks. We summarize KIF4A’s expression and functions across more than 30 malignant tumors and multiple non-neoplastic conditions—including neurodevelopmental disorders, autoimmune diseases, viral infections, fibrotic diseases, and congenital anomalies—highlighting shared molecular themes and disease-specific distinctions. A notable finding is KIF4A’s context dependency: while generally oncogenic, high KIF4A expression in cervical cancer correlates with improved survival, suggesting HPV-specific transcriptional rewiring, altered phosphorylation states, or compensatory genome stabilization as potential mechanisms.We further evaluate the translational implications of KIF4A as a biomarker for diagnosis, prognosis, and treatment response, and we critically examine therapeutic strategies targeting KIF4A—ranging from small-molecule inhibitors and gene-silencing approaches to miRNA therapeutics, exosome-based delivery systems, and neoantigen-directed immunotherapy. Finally, we outline major challenges to clinical translation, including its essential roles in mitosis and neuronal integrity, the need for tumor-selective delivery platforms, and incomplete understanding of its tissue-specific functions. Collectively, this review provides a unified mechanistic and translational framework for understanding KIF4A across human diseases, identifies key knowledge gaps, and proposes future research directions to enable safe and effective targeting of this biologically indispensable protein.

## Introduction

KIF4A, a prominent member of the kinesin superfamily, has been extensively studied for its critical roles in mitosis, including chromosome con·densation, spindle assembly, and cytokinesis [1–3]. Recent evidence reveals that KIF4A’s functions extend beyond cell division, impacting a wide array of human diseases, particularly cancers. In many malignancies, such as endometrial carcinoma [4] prostate cancer [5, 6], and glioma [7, 8], KIF4A is frequently overexpressed and contributes to tumour progression through mechanisms that enhance genomic stability, mediate therapy resistance, and modify the immune microenvironment [9–11]. These oncogenic functions underscore its central role in coordinating cell-cycle machinery with DNA repair, metabolic programs, and immune modulation. Beyond cancer, accumulating genetic and functional studies demonstrate that KIF4A also plays indispensable roles in non-neoplastic diseases, highlighting its broader biological relevance. Germline variants in KIF4A have been linked to neurodevelopmental disorders, including X-linked intellectual disability and epilepsy, where impaired axonal transport and synaptic imbalance contribute to neurological phenotypes [12–14]. Additional evidence implicates KIF4A in viral entry (HBV/HDV), autoimmune disease, fibrosis, and congenital anomalies, emphasizing that its dysregulation affects diverse physiological systems. This expanding spectrum of disease involvement illustrates the context-dependent and tissue-specific functionality of KIF4A, distinguishing it from other mitotic kinesins with more restricted biological roles.

This review aims to address these gaps by providing a comprehensive and mechanistically driven synthesis of KIF4A’s functions across human diseases. Specifically, we (1) summarize its molecular structure and regulatory network; (2) delineate its roles in cancer progression and therapy resistance; (3) integrate emerging evidence from non-neoplastic disorders to broaden the clinical relevance of KIF4A; (4) evaluate its diagnostic, prognostic, and therapeutic potential; and (5) highlight unanswered questions and future directions, including delivery strategies and context-dependent biology. By bridging molecular mechanisms with translational applications, this review offers a unified perspective that situates KIF4A as a pivotal node linking fundamental cell biology with diverse disease processes.

## Biological characteristics of KIF4A

Given KIF4A’s broad involvement across diverse pathological contexts, it is essential to first establish a clear understanding of its structural features and regulatory mechanisms. These fundamental properties form the basis for interpreting its downstream effects in both malignant and non-malignant diseases.

### Molecular structure and functional domains

KIF4A, encoded by a gene located on chromosome Xq13.1^15^, is a significant member of the kinesin superfamily. The KIF4A protein comprises a conserved N-terminal motor domain crucial for ATP hydrolysis and microtubule binding, enabling its movement along microtubules; a central α-helical stalk region; and a C-terminal tail domain that binds specific “cargo” molecules, thereby dictating its transport and functional specificity [2, 3]. Structural analyses have identified two conserved motifs within the C-terminal tail vital for chromosomal localization: a leucine zipper motif (Zip1) and a cysteine-rich motif (CR). KIF4A binds directly to the HAWK subunit NCAPG of condensin I through a conserved short linear motif (SLiM) in its tail, which relieves the autoinhibition of condensin I, significantly stimulating its ATPase activity and DNA loop extrusion capability, essential for proper chromosome condensation and segregation [1]. Deletions or mutations in these domains disrupt KIF4A’s chromosomal enrichment, leading to aberrant chromosome condensation, spindle assembly defects, and cytokinesis failure [2, 3].

### Role in the cell cycle

KIF4A’s primary functions manifest during mitosis. In interphase, KIF4A is localized in the nucleoplasm; upon nuclear envelope breakdown at mitotic entry, it rapidly associates with chromosome arms. During anaphase, it moves to the central spindle; during cytokinesis, it localizes to the contractile ring [3]. This dynamic localization underpins its multifaceted roles. First, in regulating chromosome architecture, KIF4A binds to the condensin I subunit NCAPG via its C-terminal SLiM, relieving condensin I autoinhibition and stimulating ATPase and DNA loop extrusion activities critical for chromosome condensation and segregation [1, 16]. Second, KIF4A interacts with the central spindle protein PRC1 to regulate microtubule dynamics and spindle stability [17–19]. During chromosome congregation, KIF4A and Kif18A are recruited by PRC1 to bridging fibres, where they modulate length-dependent forces at microtubule overlap zones for proper chromosome alignment. Specific inhibition of KIF4A leads to abnormal elongation of bridging fibers and chromosome misalignment, highlighting its essential role in maintaining spindle mechanical stability [18]. Moreover, KIF4A is vital for the final “abscission” step of cytokinesis. Its SUMOylation at lysine 460 (K460) enhances its affinity for the microtubule-destabilizing protein STMN1, precisely regulating microtubule dynamics for successful abscission [20].KIF4A also recruits the phosphatase PP2A-B56 complex to the central spindle, facilitating the dephosphorylation of Aurora B kinase at threonine 799 (T799), thus forming a negative feedback loop that governs anaphase microtubule dynamics and ensures the completion of cytokinesis [18, 21]. Beyond mitosis, KIF4A is involved in intracellular transport processes, notably the axonal transport of cargoes like integrin β1 in neurons, which is essential for normal neuronal development and function [22]. Additionally, during interphase, KIF4A is found in the nucleus, particularly in nucleolar regions, where it plays a role in rRNA processing and nucleolar structure maintenance. Alterations in KIF4A expression lead to disrupted nucleolar morphology and rRNA transcription efficiency, expanding its biological significance from a “mitosis-specific kinesin” to a “multifunctional protein” active throughout the cell cycle [2, 22].

### Transcriptional regulation and post-translational modifications

**Fig. 1:**
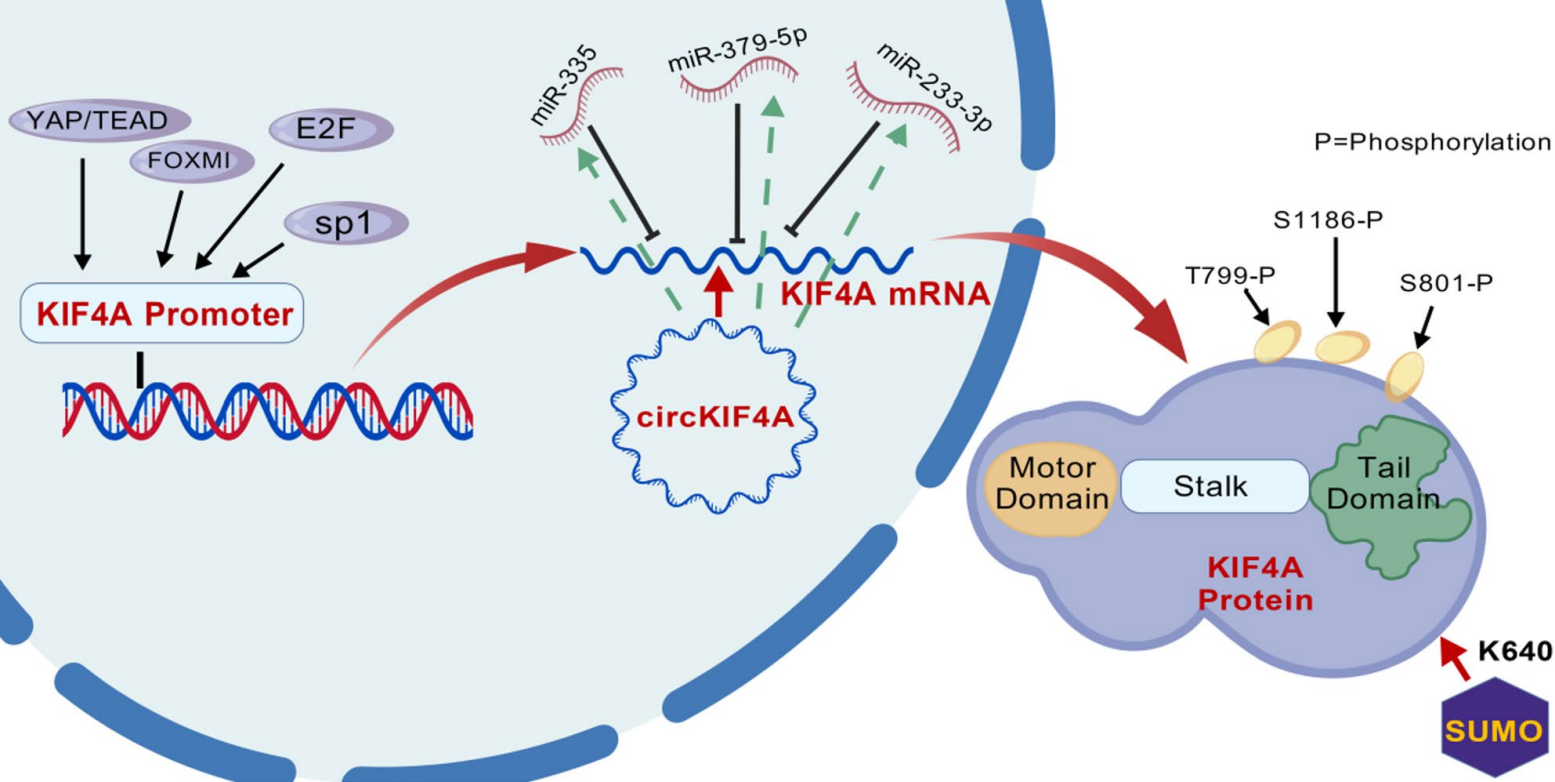
Multilevel regulatory network of KIF4A: transcriptional, post-transcriptional, and post-translational mechanisms. KIF4A is regulated at multiple levels: Transcriptional regulation: FOXM1 (in HCC), SP1 (in PCa), YAP/TEAD (in ESCC), and E2F (in multiple cancers) bind to the KIF4A promoter, thereby activating its transcription in a context-dependent manner. Post-transcriptional regulation: miRNAs (e.g., miR-335, miR-379-5p, and miR-223-3p inhibit KIF4A expression by targeting its 3′-UTR.In contrast, circKIF4A acts as a competitive endogenous RNA (ceRNA) to sponge these miRNAs, thereby alleviating the decrease and upregulating KIF4A. Post-translational modifications: Phosphorylation by CDK1 (at S1186), Aurora B (at T799), and AMPK (at S801) regulates KIF4A’s roles in chromosome condensation, spindle stability, and the metabolic coupling of mitosis. Additionally, SUMOylation at K460 regulates its role during abscission

KIF4A’s function is intricately regulated through multiple layers, forming a complex regulatory network (Fig. 1). (1) Transcriptional regulation: Key transcription factors, such as FOXM1 (notably in hepatocellular carcinoma), SP1 (in prostate cancer) [23], the YAP/TEAD complex (in oesophageal squamous cell carcinoma) [24], and E2F family members [25, 26], directly bind to the KIF4A promoter, activating its transcription. (2) Post-transcriptional regulation: Several microRNAs (miRNAs) act as negative regulators of KIF4A. For instance, miR-335, miR-379-5p, and miR-223-3p suppress KIF4A expression by targeting its 3’ untranslated region (3’-UTR), exerting tumor-suppressive effects in cancers like breast cancer [27–29]. Conversely, the circular RNA circKIF4A serves as a competitive endogenous RNA (ceRNA), sponging miRNAs such as miR-335 and miR-152, thereby alleviating their repression of KIF4A mRNA and creating a positive regulatory loop [30–32]. (3) Post-translational modifications (PTMs): These modifications play a central role in regulating KIF4A’s activity and its spatial and temporal specificity. Phosphorylation is a critical PTM, as CDK1-mediated phosphorylation at S1186 is essential for KIF4A’s association with chromosomes after nuclear envelope breakdown [11, 33]. Aurora B phosphorylation at T799 regulates its interactions with PRC1 and microtubule stabilization function [34]. Additionally, AMPK phosphorylation at S801 links cellular energy status to the regulation of the mitotic apparatus [35]. SUMOylation is another crucial modification that precisely regulates KIF4A’s function during cytokinesis [20]. Blocking KIF4A SUMOylation at K460 through CRISPR-Cas9 leads to delays in cytokinesis and defects in abscission, as SUMOylation enhances KIF4A’s binding affinity for STMN1, facilitating precise microtubule dynamics regulation [36]. studies suggest that KIF4A’s function may also be influenced by more complex mechanisms, such as liquid-liquid phase separation (LLPS), indicating that its microtubule-binding behaviour can affect other motor proteins through long-range interactions, highlighting its functional dependency on biomolecular condensate formation within the microenvironment [37].

### Mechanisms of KIF4A in cellular signalling pathways

KIF4A plays a pivotal role in regulating pathways associated with cell proliferation, including the PI3K/AKT and TGF-β/Smad pathways. In lung cancer, it promotes tumour progression through the PI3K/AKT pathway, resulting in increased expression of p21; silencing KIF4A leads to upregulation of p21 and heightened sensitivity to doxorubicin [38]. In HCC, elevated KIF4A levels correlate with enhanced Akt activity, while its knockdown reduces this signalling and induces apoptosis [39]. In breast cancer, silencing KIF4A diminishes Smad3 phosphorylation, alters TGF-β1/Smad3 pathway activity, and influences epithelial-mesenchymal transition (EMT) as well as immune factor expression [36]. In glioma, KIF4A enhances the expression of BUB1, activating the TGF-β/SMAD pathway, which promotes EMT and malignant progression [40, 41].

In terms of metabolic regulation, KIF4A facilitates glycolytic reprogramming [42]. In colorectal cancer (CRC), it enhances cancer stem cell (CSC) properties and resistance to chemotherapy by promoting glycolysis, including the upregulation of related proteins and increased lactate production; inhibition of glycolysis reverses this phenotype [43]. TheCircKIF4A, by sponging miR-335 in breast cancer and glioma, derepresses ALDOA and OCT4, increases HK2/PKM2 expression, and drives glycolysis and metastasis [30, 31].

KIF4A also forms a critical positive feedback loop with the Hippo-YAP pathway. In ESCC, YAP/TEAD4 directly activates KIF4A transcription, stimulating proliferation and migration [24]. In urothelial bladder cancer (UBC), YAP1 promotes KIF4A transcription, while KIF4A inhibits YAP1 phosphorylation, facilitating its nuclear translocation and activation, thus creating a self-amplifying oncogenic circuit [44]. In osteosarcoma, the DEPDC1/KIF4A axis promotes malignant behaviours and EMT by inhibiting the Hippo pathway, resulting in decreased p-LATS1/p-YAP levels [45].

Moreover, KIF4A is integral to the DNA damage response (DDR). In CRC, it interacts directly with core proteins of homologous recombination repair (HRR) such as RAD51 and BRCA2 through its motor and tail domains. It is specifically phosphorylated by Cdk1 and Aurora B during mitosis, modifications that may influence its interaction with condensin I and its recruitment to DNA break sites, thereby enhancing HRR and contributing to therapeutic resistance [10]. In stomach adenocarcinoma (STAD), KIF4A is co-upregulated with HRR genes like RAD51, exacerbating genomic instability [42].

**Fig. 2:**
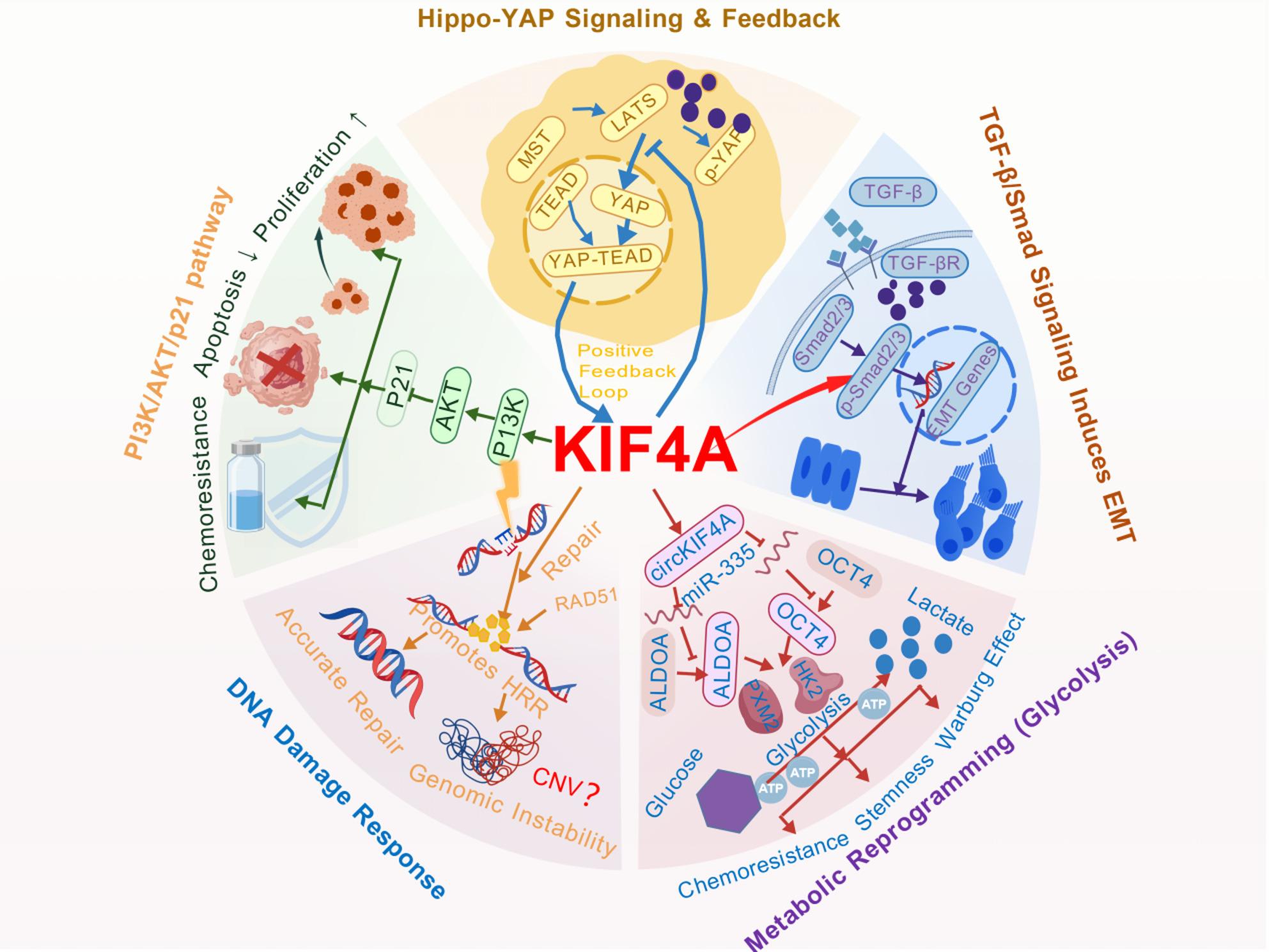
KIF4A modulates a central network of oncogenic signaling pathways. KIF4A functions as a multifaceted hub orchestrating essential processes in tumorigenesis by engaging with five major signaling axes. It activates PI3K/AKT signalling while suppressing p21, thus promoting proliferation and chemoresistance [38]. KIF4A also inhibits LATS kinase to allow YAP nuclear translocation and transcription; in turn, nuclear YAP/TEAD complexes enhance KIF4A expression, creating a positive feedback loop [24, 44]. Furthermore, KIF4A amplifies TGF-β/Smad signaling to facilitate EMT [36, 40, 41]. Through circKIF4A-mediated sequestration of miR-335, KIF4A elevates ALDOA and OCT4 levels, enhancing glycolytic flux and stemness [30, 31, 43]. Additionally, KIF4A supports homologous recombination repair via its motor and tail domains, which increases genomic instability and resistance to DNA-damaging agents [11, 42]. Symbols: →, activation; ⊥, inhibition; P, phosphorylation

In conclusion, KIF4A is intricately involved in multiple core signaling pathways—PI3K/AKT, TGF-β/Smad, Hippo-YAP, metabolism, and DDR—through cross-interactions (Fig. 2). This establishes its multifunctionality, highlights its role as a central hub within signaling networks, and supports the rationale for combined therapeutic strategies targeting KIF4A and its associated pathways.

## Role of the target molecule in disease pathogenesis and progression

Before discussing disease-specific roles of KIF4A, it is important to note that most malignant phenotypes converge on a small set of biological processes—cell cycle control, DNA repair, metabolic rewiring, immune evasion, and EMT. These shared mechanisms help contextualize its functions across different cancer types.

### Expression and function in malignancies

In **breast cancer (BC)**, KIF4A is a prominent oncogenic driver, exhibiting significant upregulation that correlates with tumour malignancy and poor prognosis, demonstrating predictive power that surpasses traditional clinical indicators [15, 29, 46]. Its regulation involves non-coding RNA networks: circKIF4A sponges miR-335 to promote glycolysis and liver metastasis [31], while also sponging miR-152 to facilitate EMT and inhibit apoptosis [32]. In triple-negative BC (TNBC), circKIF4A interacts with EIF4A3 to stabilize SDC1, activating the c-Src/FAK pathway [47], and additionally sponges miR-375, creating a positive feedback axis [48]. miR-379-5p and miR-223-3p negatively regulate KIF4A; their inhibition reduces malignant phenotypes and induces apoptosis [27, 28]. KIF4A is implicated in tamoxifen resistance (regulated by ANCCA/MLL) [49], serves as a hub gene within EMT-related ceRNA networks [50], and maintains CSC properties (its inhibition diminishes tumour sphere formation capacity [47, 51]. Co-expression with the PTTG family regulates cell cycle and metabolism, with high KIF4A levels predicting poor prognosis [52]. Its overexpression can inhibit PARP-1 activity, enhancing doxorubicin-induced apoptosis [53], and it influences the immune microenvironment via TGF-β1/Smad3^36^. It also interacts with ERCC6L to promote metastasis [54]. Furthermore, KIF4A is incorporated into multiple prognostic models (5-gene immune signature [55], 8-gene hypoxia model [56], 6-KIF risk model [57], E2F target gene signature [58]) and is associated with taxane resistance [59]. Weighted Gene Co-expression Network Analysis (WGCNA) revealed that it is as a hub gene that modulates distant metastasis (enriched in EMT pathways [60]); involved in the regulation of subnetwork, thereby accurately distinguishing BC from normal tissue [61]; and it correlates with APOBEC mutational signature 2, potentially influencing DNA damage repair [62]. Collectively, these studies underscore KIF4A’s crucial role in BC diagnosis, prognosis, and therapy.

In **endometrial cancer (EC)**, KIF4A expression is markedly increased and closely linked to poor prognosis [4, 63]. Its primary mechanism involves direct interaction with TPX2, inhibiting its ubiquitination and degradation, thus enhancing TPX2 stability and helping EC cells maintain genomic integrity under replication stress [4]. Functional studies indicate that KIF4A knockdown triggers DNA damage response, cell cycle arrest, and apoptosis, significantly curtailing tumour proliferation [4, 64]. KIF4A also correlates with the mRNA stemness index (mRNAsi), suggesting a role in supporting EC cancer stem cell characteristics [64]. Furthermore, high KIF4A expression is associated with advanced FIGO stage, high tumour grade, specific TCGA molecular subtypes, and the presence of various tumour-infiltrating immune cells, indicating its profound role in regulating the EC immune microenvironment [63]. These findings affirm KIF4A’s oncogenic function in EC, positioning it as a potential prognostic marker and therapeutic target [4, 63, 64].

In **prostatic cancer (PCa)**, KIF4A levels are significantly elevated, particularly in castration-resistant PCa (CRPC) and advanced stages. High expression is linked to reduced overall survival (OS), PSA failure, and shorter biochemical recurrence (BCR)-free survival, establishing it as an independent predictor of BCR [6, 65]. The key mechanisms include the formation of a positive feedback loop between KIF4A and AR/AR-V7, which prevents their degradation via ubiquitination; AR transcriptionally activates KIF4A, driving CRPC progression and enzalutamide resistance, which can be reversed by KIF4A knockdown [5].Furthermore, SP1 activates KIF4A transcription, and KIF4A associates with TWIST1 to promote proliferation, invasion, and EMT [23]. KIF4A is featured in several prognostic models, including a progression-free interval model with UBE2C [66], a 5-gene OS model [67], and a 9-gene BCR risk model [46]), with the latter outperforming traditional indicators. These studies affirm KIF4A’s significance as a prognostic marker and therapeutic target in PCa [5, 6, 46, 65–67].

In **bladder cancer (BC)**, KIF4A contributes to disease progression by modulating the tumour immune microenvironment [9]. Research reveals that KIF4A is highly expressed in high-grade BC and correlates with reduced CD8 + T cell infiltration and poor prognosis [9, 68]. Mechanistically, KIF4A enhances the expression of the chemokine CXCL5, which recruits myeloid-derived suppressor cells (MDSCs) to the tumour microenvironment, thereby inhibiting antitumor immunity, facilitating immune evasion, and promoting tumour growth [9]. Consequently, KIF4A has been identified as an independent prognostic predictor in BC^68^, and targeting the KIF4A/CXCL5/MDSC axis may represent a therapeutic strategy to enhance the efficacy of immunotherapy [9].

In **glioma**, KIF4A is a crucial oncogene, markedly overexpressed and significantly linked to poor patient prognosis and reduced survival [7, 8, 69]. Mechanistically, KIF4A promotes the reorganization of the actin cytoskeleton by transcriptionally repressing Rac1 and Cdc42, thereby enhancing tumour cell proliferation, invasion, and migration [7]. It also upregulates BUB1, which activates the TGF-β/SMAD pathway, promoting EMT, and affecting glioma stem cells (GSCs) and their associated signalling, which contributes to malignant progression [40, 41]. KIF4A is part of a five-KIF prognostic signature, with its high expression correlating with IDH1 wild-type status and upregulated immune checkpoints activity [70]. The small molecule inhibitor WZ-3146 targets KIF4A, suppressing glioma cell proliferation and inducing apoptosis in vitro via Caspase-3 activation, positioning it as a potential candidate for clinical application [8].

In **hepatocellular carcinoma (HCC)**, KIF4A is significantly elevated, with high levels associated with adverse clinicopathological features and reduced survival [39, 71–73]. Mechanistically, the transcription factor FOXM1, particularly its FOXM1c isoform, binds directly to the KIF4A promoter to enhance its transcription, establishing a regulatory axis between FOXM1 and KIF4A. KIF4A serves as a key downstream effector in FOXM1-induced HCC cell proliferation; silencing KIF4A negates the pro-tumorigenic effects of FOXM1 overexpression [74]. Moreover, KIF4A forms a co-expression network with CDCA5, TPX2, FOXM1, ASF1B, and AURKB, which collectively regulate cell cycle progression and promote HCC development [75–77]. Hypomethylation of its promoter region may also contribute to its upregulation [73].

In **colorectal cancer (CRC)**, elevated KIF4A expression is positively associated with poor tumour regression following neoadjuvant chemoradiotherapy (nCRT) [10, 78]. Mechanistically, KIF4A is involved in the chemotherapy-induced DNA damage response (DDR) through its motor and tail domains, enhancing CRC cell chemoresistance [10]. It also promotes glycolytic metabolism by upregulating related proteins and increasing lactate production, thus enhancing CSC properties and chemoresistance; inhibiting glycolysis can reverse this resistant phenotype [43]. Furthermore, KIF4A neoantigens may activate the immune response, particularly in CRC subtypes with high immune gene expression, suggesting that vaccines targeting these neoantigens could improve survival [79].

In **esophageal squamous cell cancel (ESCC)**, KIF4A expression is significantly increased and associated with poor prognosis [80, 81]. Mechanistically, its transcription is directly activated by the Hippo pathway effector YAP/TEAD4, promoting cell proliferation, migration, and inhibiting apoptosis [24]. Epigenetic changes, such as the downregulation of EZH2, lead to reduced H3K27me3 modification on the KIF4A promoter, thereby derepressing its expression [82]. Additionally, ATAD2 positively regulates KIF4A; their co-expression fosters malignant progression, and aspirin can inhibit ESCC cell phenotypes by downregulating the ATAD2/KIF4A axis [83]. Diagnostically, the combination of KIF4A, RAD51AP1, and CDKN3 shows exceptionally high diagnostic efficacy for ESCC (AUC = 0.979) [84]. Epidemiological studies indicate that environmental exposures, such as chemical burns to the esophagus, may promote carcinogenesis by upregulating multiple cell cycle core genes, including KIF4A (e.g., TOP2A, CCNB1), providing a unique perspective on its role in cancer initiation [85].

In **stomach adenocarcinoma (STAD)**, KIF4A is closely linked to the homologous recombination repair (HRR) pathway and is co-upregulated with core HRR genes like RAD51^42^. Cytoplasmic circKIF4A acts as a ceRNA, sponging miR-144-3p to derepress EZH2, promoting EMT and proliferation [86]. KIF4A overexpression contributes to genomic instability, such as a high CNV burden), and is associated with reduced immune cell infiltration [42], enhanced CSC properties, and chemoresistance [87, 88]. Therefore, KIF4A may serve as a potential predictive marker of poor prognosis [89] and target for combination therapy with DNA-damaging agents or immunotherapy in STAD. DEPDC1 interacts with KIF4A, and their co-expression promotes progression; knocking down DEPDC1 activates the Hippo pathway, inhibiting osteosarcoma (OS) malignant behaviour, while overexpressing KIF4A reverses this effect, indicating that the DEPDC1/KIF4A axis promotes OS progression by inhibiting the Hippo pathway [45]. Additionally, exosomes from normal human chondrocytes deliver miR-195 to OS cells, targeting the KIF4A 3’UTR to inhibit its expression, resulting in a about 40% reduction in tumour volume in vivo and a marked decrease in OS growth [90].

In **lung cancer**, KIF4A plays a pivotal role in tumour progression and drug resistance by regulating various signalling pathways and mediating drug efflux. It modulates the PI3K/AKT pathway and downstream p21 expression; silencing KIF4A leads to increased p21 levels, inhibited proliferation, and enhanced sensitivity to doxorubicin in A549 cells [38]. Its expression correlates with neuronal guanine nucleotide exchange factor (NGEF), promoting neural infiltration through the Ephrin-A3/EphA2 axis [91]. By forming a functional complex with PHF14, KIF4A synergistically facilitates lung cancer cell proliferation [92]. Utilizing its tail domain, KIF4A interacts with the N-terminus of lung resistance-related protein (LRP), facilitating the transport of LRP-positive vault complexes along microtubules to the cell membrane, thereby promoting drug efflux and enhancing chemoresistance [93, 94]. KIF4A is identified as a core lysosome-related genes, forming a prognostic signature with CCNA2, DLGAP5, among others, involved in regulating glucose metabolism, amino acid metabolism, and immune response; its overexpression significantly correlates with poor patient prognosis [78].

In **clear cell renal cell carcinoma (ccRCC)**, KIF4A is significantly overexpressed. Its expression correlates with advanced clinical stage, higher tumour grade, shorter overall survival, and specific immune infiltration patterns, such as increased M2 macrophage infiltration [95–97]. Mechanistically, KIF4A likely drives ccRCC progression through two main pathways: regulating the cell cycle and remodelling the tumour immune microenvironment, establishing it as a potential prognostic biomarker associated with immune infiltration [95, 98].

In **ovarian cancer (OC)**, KIF4A is highly expressed, and its knockdown effectively inhibits OC cell proliferation, migration, and induces apoptosis [99, 100]. Mechanistically, KIF4A directly binds and positively regulates the mitotic checkpoint protein BUB1, thereby forming an oncogenic axis [99]. Additionally, the nuclear import protein KPNA2 acts as an upstream regulator; its knockdown results in decreased KIF4A protein levels, suggesting that KPNA2 promotes OC progression by upregulating KIF4A signalling [100], which positions KIF4A as a potential therapeutic target in OC.

In **laryngeal squamous cell carcinoma (LSCC)**, KIF4A is significantly overexpressed and associated with poor patient prognosis [101]. Its expression is regulated by the ERCC6L-FOXM1 signalling axis, where ERCC6L facilitates the nuclear translocation of FOXM1, enabling it to directly bind to the KIF4A promoter and activate transcription. The resultant increase in KIF4A drives LSCC cell proliferation and migration while inhibiting apoptosis; conversely, KIF4A knockdown mitigates the pro-tumour effects of ERCC6L overexpression [101]. This signalling axis may serve as a promising target for LSCC therapy.

In **urothelial bladder cancer (UBC)**, KIF4A establishes a positive feedback loop with the Hippo pathway core molecule YAP1, collaboratively driving tumour progression. KIF4A levels are elevated in UBC and predict poor prognosis; its knockdown inhibits cell proliferation, migration, invasion, and EMT, while inducing apoptosis and suppressing AKT signalling [44]. Mechanistically, YAP1 enhances KIF4A transcription, while KIF4A inhibits YAP1 phosphorylation, promoting its nuclear translocation and activation, thereby sustaining oncogenic signalling activation [44]. This finding underscores the central role of KIF4A in UBC.

In **cholangiocarcinoma (CCA)**, KIF4A is significantly upregulated, and its elevated expression correlates with poorer overall survival in patients [102]. Mechanistically, KIF4A levels are associated with the infiltration of activated memory T cells and mast cells in the tumour microenvironment, suggesting its potential role in influencing tumour progression through the modulation of immune cell infiltration and tolerance [102]. This study indicates KIF4A as a novel biomarker for predicting CCA prognosis and guiding immunotherapy strategies.

In **oral squamous cell carcinoma (OSCC)**, KIF4A is significantly upregulated in both cells and tissues, correlating with tumour size [103]. Mechanistically, KIF4A upregulates CCL2, which binds to the CCR2 receptor on macrophages, facilitating their recruitment and polarization towards the pro-tumour M2 phenotype [104]. Additionally, KIF4A knockdown activates the spindle assembly checkpoint (SAC), evidenced by increased localization of BUB1 and MAD2 at the centromere, downregulation of CDC20, and accumulation of Cyclin B1, resulting in G2/M phase arrest and inhibition of cell proliferation [103]. KIF4A thus serves as a key regulator of OSCC tumour progression and represents a potential therapeutic target.

**Pancreatic ductal adenocarcinoma (PDAC)**: KIF4A is part of a 36-gene prognostic signature for PDAC, with its elevated expression correlating with reduced patient survival [105]. This signature has been validated across multiple independent clinical cohorts, demonstrating predictive capabilities that surpass traditional TNM staging and tumour grading. Furthermore, it serves as an independent prognostic factor, aiding in clinical risk assessment and treatment planning for PDAC patients.

**Special Finding**: In the context of **cervical cancer**, KIF4A displays a distinct role that contrasts with its typically oncogenic function in other cancers, highlighting its context-dependent behaviour. Genetically, KIF4A is upregulated due to amplification at Xq12^106^, and shows high expression in HPV16-positive cervical cancer, where it is enriched in pathways related to the cell cycle and DNA repair, potentially under the regulation of E2F [107]. KIF4A is recognized as a key player in the progression of cervical cancer, possibly promoting disease advancement by modulating the cell cycle [108]. It is also part of a co-upregulated gene cluster alongside KIF11, KIF14, and PRC1, which collectively mediates cytokinesis and participates in disrupted mitotic gene networks [109, 110]. However, functional studies yield contradictory evidence: high KIF4A expression is associated with improved OS and progression-free survival (PFS) in cervical cancer patients, with experiments demonstrating its ability to inhibit cell proliferation and migration while inducing G1 phase arrest, characteristics typical of tumour suppressor genes [111]. Notably, the low-expression cohort exhibits a higher CNV burden and an increased incidence of NOTCH1 mutations, suggesting they may benefit more from immunotherapy [111]. This functional dichotomy could be linked to the tumour microenvironment associated with high-risk HPV infections and the specificities of cervical tissue; further exploration of the underlying mechanisms is necessary, as clinical applications hinge on precise molecular subtyping [106–109, 111].

**Possible Mechanisms Underlying the Tumor-Suppressive Role of KIF4A in Cervical Cancer**: Although KIF4A generally functions as an oncogene across multiple malignancies, accumulating evidence indicates that in cervical cancer it exhibits a paradoxical tumor-suppressive behavior, with high KIF4A expression correlating with improved OS and PFS and experimental data showing reduced proliferation and migration upon its overexpression. This functional divergence suggests a unique regulatory landscape within HPV-associated cervical epithelium. Several biologically plausible and testable hypotheses may explain this context-dependent role [106–109]. (1) First, HPV-driven E2F hyperactivation may fundamentally reshape the KIF4A interactome. High-risk HPV (especially HPV16) induces constitutive degradation of p53 and Rb via E6/E7 oncoproteins, resulting in persistent activation of E2F transcription. While E2F typically upregulates KIF4A to promote proliferation, the unusually high E2F activity in HPV-positive cells may bias KIF4A toward engaging stabilizing partners—such as PRC1 and condensin I—rather than oncogenic effectors like BUB1 or glycolytic regulators. This shift could enhance genomic stability and buffer virus-induced mitotic stress. Testable prediction: protein-interaction profiling will show preferential KIF4A–PRC1/condensin I binding in HPV16 cells [110].(2) HPV infection may alter the phosphorylation landscape of KIF4A, reprogramming its function. KIF4A activity is regulated by phosphorylation at key residues (Aurora B T799, CDK1 S1186, AMPK S801). HPV-driven mitotic deregulation may modify these phosphorylation events, promoting chromosome condensation and spindle stability at the expense of proliferative signaling. Testable prediction: HPV-positive cervical cancer cells display a distinct pattern of Aurora B/CDK1-dependent KIF4A phosphorylation detectable by phosphoproteomics.(3) KIF4A upregulation may act as a compensatory genome-stabilizing mechanism in a microenvironment characterized by high chromosomal instability (CIN). HPV infection significantly increases mitotic errors, anaphase bridges, and DNA damage. As a master regulator of chromosome condensation and midzone mechanics, KIF4A may reduce catastrophic mitotic failure by enforcing stricter genomic maintenance. Tumors with high KIF4A expression may therefore exhibit lower CIN burden, slower evolutionary dynamics, and better clinical outcomes. Testable prediction: KIF4A-high tumors display fewer CIN signatures (e.g., micronuclei, chromosomal bridges) than KIF4A-low counterparts [111].(4) immune-microenvironment differences may contribute to the divergent prognosis. Recent studies show that KIF4A-low cervical cancers exhibit greater CNV burden and enrichment of NOTCH1 mutations—features associated with increased neoantigen load and immunotherapy sensitivity. Conversely, KIF4A-high tumors may maintain more intact genomic architecture and a less immunosuppressive microenvironment, resulting in slower progression. Testable prediction: KIF4A-high tumors exhibit lower levels of T-cell exhaustion markers and fewer immunosuppressive cytokines [112].(5) cervical epithelium expresses a unique cluster of mitotic regulators, such as KIF11, KIF14, and PRC1, which are consistently co-upregulated with KIF4A. This coordinated module may prioritize accurate chromosome segregation rather than aggressive proliferation. Testable prediction: co-expression networks in cervical cancer will reveal a stronger KIF4A–KIF11–PRC1 regulatory axis compared with other malignancies.

Additionally, the aberrant expression and function of KIF4A have been implicated in the pathogenesis and progression of a variety of solid tumours, including **insulinoma** [82], **pleural mesothelioma (PM)** [112], **meningioma** [113], **skin cutaneous melanoma** (SKCM) [114], and **Ewing sarcoma (ES)** [115]. Collectively, these studies identify KIF4A as a pivotal oncogene with broad implications across different cancers, influencing malignant progression through its regulation of diverse biological processes such as the cell cycle, metabolism, apoptosis, EMT, and the immune microenvironment.

### Pan-cancer characteristics of KIF4A

Although KIF4A exhibits cancer-type-specific mechanisms, these individual observations converge into several shared molecular patterns across malignancies. Therefore, a pan-cancer perspective is crucial to understand the conserved pathways through which KIF4A drives tumorigenesis.KIF4A demonstrates significant overexpression in 18 different malignant tumours (including breast cancer, HCC, ccRCC, CRC, oesophageal cancer, etc.) ^116^, and exhibits the following core features at a pan-cancer level: ① Convergent regulatory pathways: KIF4A is primarily enriched in pathways related to the cell cycle, DNA replication, and immune regulation, often forming co-expression networks with genes such as CDCA5, TPX2, and FOXM1^75,117^. ② Conserved key regulatory axes: The FOXM1-KIF4A axis observed in HCC [74] and lung adenocarcinoma [118]) and the YAP/TEAD-KIF4A axis (noted in ESCC [24] and UBC [44]) exert pro-tumorigenic effects across multiple cancer types. ③ Consistent clinical significance: High expression levels correlate with advanced tumour stages, higher pathological grades, and poor patient prognosis, indicating its potential as a molecular marker for pan-cancer prognostic evaluation [116, 119].

### Expression and function in non-neoplastic diseases

While KIF4A is best known for its oncogenic functions, recent studies demonstrate that its biological impact extends well beyond cancer. The following section summarizes its roles in neurodevelopmental, immune-mediated, infectious, and metabolic disorders, underscoring its broad clinical relevance.In **neurodevelopmental disorders**, variants in the KIF4A gene are linked to a range of congenital genetic diseases. Whole-exome sequencing and linkage analyses in multiple affected families have identified pathogenic mutations in KIF4A (including missense mutations such as p.Leu533Phe, p.Asp371His, and p.Arg771Lys, as well as splice site, frameshift, and indel mutations) [12–14, 120, 121]. These mutations lead to global developmental delays, intellectual disabilities, brain structural abnormalities (such as hydrocephalus), and multi-organ congenital anomalies, collectively referred to as X-linked Intellectual Disability 100型 (XLID100) [12, 13]. Additionally, duplications in the Xq13.1 region containing KIF4A have been identified in patients with floppy infant syndrome (FIS), suggesting that abnormalities in KIF4A dosage may be linked to this condition [122]. The pathogenic mechanism involves compromised KIF4A protein function, which is essential for intracellular transport, neuronal survival, ciliogenesis, and the cell cycle [12, 121]. Reducing KIF4A expression in primary rat hippocampal neurons disrupts the balance between excitatory and inhibitory synaptic transmission, providing a neurobiological explanation for the intellectual disabilities and epilepsy observed in affected individuals [121]. These findings underscore KIF4A’s critical role in the development of the nervous system and offer a genetic basis for diagnosing congenital anomalies.

In **neuropsychiatric disorders**, mutations in the KIF4A gene are closely linked to the pathogenesis of epilepsy. A specific point mutation (R728Q) enhances KIF4A’s affinity for PARP1, disrupting the PARP1-TrkB-KCC2 signalling pathway and resulting in abnormal morphology of dendrites and spines in hippocampal neurons, significantly lowering the seizure threshold. Animal studies indicate that NAD supplementation can ameliorate the mutant phenotype, positioning KIF4A or PARP1 as potential intervention targets for epilepsy [123]. Moreover, a functional deficiency in KIF4A is associated with intellectual disabilities, as mutations disrupt the balance of excitatory-inhibitory synaptic transmission, leading to neurodevelopmental delays and cognitive impairments [121].

In **autoimmune and fibrotic diseases**, KIF4A exhibits diverse functional characteristics. In autoimmune conditions, anti-KIF4A antibodies have been linked to ANCA-associated vasculitis (AAV): antigen microarray screening revealed a significantly higher prevalence of anti-KIF4A antibodies among AAV patients compared to healthy controls and individuals with other autoimmune diseases, suggesting its potential as a novel serological diagnostic marker for AAV, warranting further investigation to validate its specificity and clinical utility [124]. In mechanisms underlying immune comorbidities, KIF4A serves as a shared hub gene in both psoriasis and Crohn’s disease, enriched in cell cycle and immune pathways; treatments like etoposide can ameliorate comorbid progression by downregulating its expression, indicating KIF4A’s role in these interconnected mechanisms [125]. In fibrotic diseases, KIF4A is a critical gene implicated in idiopathic pulmonary fibrosis (IPF), showing upregulation in pulmonary fibrosis models; the traditional Chinese medicine formula Jinshui Huanxian (JHF) can reverse its aberrant expression and improve fibrotic pathology, highlighting its potential as a therapeutic target in IPF [126].

In **infectious diseases**, KIF4A is pivotal for the entry of viral hepatitis pathogens. Its ATPase activity facilitates interaction with the cellular surface receptor NTCP for HBV and HDV, promoting NTCP’s transport along microtubules to the cell membrane, enhancing the efficiency of viral attachment and infection. This finding reveals a novel mechanism through which viruses exploit host kinesins to facilitate infection, providing a foundation for developing anti-viral strategies targeting KIF4A [127]. In the context of HCC pathogenesis, HBV infection can elevate KIF4A expression by activating its promoter, a mechanism that may significantly contribute to the development of HBV-associated HCC development [128].

In **connective tissue diseases**, preliminary studies have suggested a potential link between KIF4A and the pathological processes underlying classical Ehlers-Danlos syndrome (cEDS). Altered expression of KIF4A has been observed in skin fibroblasts from cEDS patients, indicating a possible role in extracellular matrix (ECM) remodelling or maintenance of endoplasmic reticulum homeostasis, although specific mechanisms require further exploration [129].

In **dental diseases**, mutations in KIF4A have been associated with particular tooth morphogenesis defects. Linkage analysis and exome sequencing in two families with X-linked dental anomalies (including taurodontism, microdontia, and dens invaginatus) have identified two independent pathogenic missense variants in KIF4A [120]. Functional experiments indicate that these variants disrupt cell migration, and KIF4A is expressed during tooth development; its dysfunction results in tooth shape abnormalities, providing a genetic basis for diagnosis and counselling [120].

In **reproductive and developmental system diseases**, mutations in KIF4A can lead to male infertility and early embryonic developmental issues. These mutations impact sperm centrosome integrity and spindle stability, causing fertilization failures and embryonic developmental arrests, thus serving as a basis for genetic diagnosis in cases of unexplained male infertility and informing assisted reproductive strategies [130]. Moreover, KIF4A variants are associated with severe fetal anomaly syndromes, underscoring its important role in early embryonic mitosis and cell migration [131]. Furthermore, KIF4A expression is diminished in intrauterine adhesion (IUA) lesions, suggesting its involvement in IUA pathogenesis by inhibiting endometrial cell mitosis and regeneration [132].

Beyond its role in distinct diseases, KIF4A is pivotal in disease comorbidity mechanisms. Notably, in the context of metabolic abnormalities and cancer, type 2 diabetes (T2D) is recognized as a risk factor for malignancies such as HCC and breast cancer. KIF4A serves as a common gene between these conditions, suggesting a potential link between T2D-related metabolic disorders and the malignant phenotypes of tumours through shared signalling pathways, including p53 and MAPK. This connection presents KIF4A as a promising combined intervention target for patients with T2D complicated by cancer [133]. Furthermore, KIF4A exhibits differential expression in tissues affected by COVID-19 and those with digestive system cancers, highlighting its involvement in cell division pathways and offering fresh insights into the mechanisms underlying the interaction between infection and cancer [134]. KIF4A dysfunction is also closely associated with the pathogenic processes induced by environmental toxins. Research indicates that the environmental pollutant bisphenol A (BPA) can activate the estrogen-related receptor (esERR)-KIF4A (esKIF4A) signalling pathway, resulting in reduced testis weight, abnormal spermatogenesis, and increased apoptosis in the Chinese mitten crab (Eriocheir sinensis). This underscores KIF4A’s critical role in mediating the reproductive toxicity of environmental chemicals [77].

To provide a comprehensive overview of KIF4A’s expression, function, and mechanisms across various diseases, we have compiled the following table (Table 1). This table details KIF4A’s expression status, core functions, associated molecular mechanisms, and clinical significance across a range of diseases, aiming to clarify KIF4A’s complex roles in diverse pathological conditions and its potential as both a biomarker and a therapeutic target.

**Table 1:** Expression, Function, and associated mechanisms of KIF4A in various diseases

Disease Category	Disease Name	Core Mechanism of Action	Key Signalling Pathways/Interacting Molecules	Expression/Significant Features	Biological Effects	Clinical Relevance	Ref.
Cancer	Breast Cancer (BC)	circKIF4A sponges miRNAs to promote glycolysis/EMT/metastasis; 2. Maintains CSC traits; 3. Involved in tamoxifen resistance	circKIF4A/miR-335/ALDOA, KIF4A-ERCC6L	Overexpression, correlates with malignancy, taxane resistance; included in multiple prognostic models	Promotes proliferation, migration, resistance; knockdown induces apoptosis	Diagnostic/prognostic marker; target for TNBC and resistance	[27, 31, 51, 53, 54]
	Endometrial Cancer (EC)	Interacts with TPX2 to inhibit its degradation, maintaining genomic stability; modulates immune microenvironment	KIF4A/TPX2 axis	Overexpression, associated with advanced FIGO stage, specific TCGA subtypes	Promotes mitosis; knockdown causes DNA damage, cell cycle arrest	Poor prognostic marker; potential therapeutic target	[4, 63, 64]
	Prostate Cancer (PCa/CRPC)	Forms positive feedback loop with AR/AR-V7 promoting castration resistance; SP1/KIF4A/TWIST1 promotes EMT	AR/KIF4A feedback, SP1/KIF4A/TWIST1	Overexpression in CRPC, associated with BCR; included in prognostic models	Promotes proliferation, EMT, resistance; knockdown reverses enzalutamide resistance	Independent predictor of BCR; target for CRPC	[5, 6, 23, 65]
	Bladder Cancer (non-urothelial)	It upregulates CXCL5 to recruit MDSCs, inhibiting CD8⁺ T cells	KIF4A/CXCL5/MDSC axis	Overexpression in high-grade tumours, correlates with reduced CD8⁺ T cells and poor prognosis	Promotes immune evasion, tumour growth	Prognostic marker; potential target for combination immunotherapy	[9, 68]
	Glioma	Suppresses Rac1/Cdc42 to promote invasion; activates TGF-β pathway; targeted by WZ-3146	KIF4A/Rac1/Cdc42, KIF4A/BUB1/TGF-β	Overexpression, associated with shorter survival	Promotes proliferation, invasion; WZ-3146 induces apoptosis	Prognostic indicator; WZ-3146 as candidate drug	[7, 8, 40, 70]
	Hepatocellular Carcinoma (HCC)	FOXM1 activates KIF4A; forms co-expression network with CDCA5/TPX2	FOXM1-KIF4A, CDCA5-KIF4A-TPX2	Overexpression, correlates with tumour size, vascular invasion	Promotes cell cycle, proliferation; knockdown abrogates FOXM1 oncogenic effects	Prognostic marker; potential target	[71–76]
	Colorectal Cancer (CRC)	Regulates DDR to promote resistance; enhances glycolysis to boost CSC traits	DDR pathway, glycolytic pathway	Overexpression, associated with nCRT resistance	Promotes resistance, CSC traits; glycolysis inhibition reverses resistance	Predictive marker for nCRT response; combination target	[10, 43, 79]
	Oesophageal Squamous Cell Carcinoma (ESCC)	YAP/TEAD activates KIF4A; ATAD2 cooperatively promotes cancer; aspirin downregulates its expression	YAP/TEAD-KIF4A, ATAD2/KIF4A	Overexpression; diagnostic AUC = 0.979 with RAD51AP1/CDKN3	Promotes proliferation, migration; inhibits apoptosis	Diagnostic/prognostic marker; aspirin target	[24, 81, 83, 84]
	Stomach Adenocarcinoma (STAD)	Cooperates with RAD51 causing genomic instability; circKIF4A promotes EZH2 expression	HRR pathway, circKIF4A/miR-144-3p/EZH2	Overexpression, associated with high CNV burden, reduced immune infiltration	Promotes immune evasion, resistance; impairs DNA repair	Prognostic marker; target for combination with DDR/immunotherapy	[42, 86, 89]
	Osteosarcoma (OS)	DEPDC1/KIF4A inhibits Hippo pathway; exosomal miR-195 targets KIF4A	DEPDC1/KIF4A-Hippo, miR-195-KIF4A	Co-overexpression with DEPDC1; target of miR-195	Promotes proliferation, EMT; miR-195 suppresses expression, reducing tumour volume by 40%+	DEPDC1/KIF4A as nodal point; exosomal target	[45, 90]
	Lung Cancer	Regulates PI3K/AKT/p21; interacts with LRP to promote drug efflux	PI3K/AKT/p21, KIF4A-LRP	Overexpression, associated with cisplatin/doxorubicin resistance	Promotes proliferation; knockdown enhances chemosensitivity	Chemosensitization target; prognostic marker	[38, 92 ,93, 118]
	Skin Cutaneous Melanoma (SKCM)	Promotes proliferation, migration; reduces CD8⁺ T cell infiltration	Immune infiltration pathways	Overexpression, associated with poor prognosis	Promotes immune evasion	Prognostic marker; potential guide for immunotherapy	[114]
	Clear Cell Renal Cell Carcinoma (ccRCC)	Regulates cell cycle; increases M2 macrophages	Cell cycle pathways, immune pathways	Overexpression, associated with advanced stage, M2 macrophage infiltration	Promotes proliferation; modulates immune infiltration	Immune-related prognostic marker	[95, 96, 98]
	Ovarian Cancer (OC)	Interacts with BUB1; KPNA2 upregulates its expression	KIF4A/BUB1, KPNA2/KIF4A	Overexpression, associated with poor prognosis	Promotes proliferation, migration; inhibits apoptosis	Potential therapeutic target	[99, 100]
	Laryngeal Squamous Cell Carcinoma (LSCC)	ERCC6L-FOXM1 activates KIF4A transcription	ERCC6L-FOXM1-KIF4A	Overexpression, associated with poor prognosis	Promotes proliferation, migration; inhibits apoptosis	Prognostic indicator; potential intervention node	[101]
	Urothelial Bladder Cancer (UBC)	Forms positive feedback loop with YAP1	KIF4A/YAP1 positive feedback	Overexpression, associated with poor prognosis	Promotes proliferation, EMT; inhibits apoptosis	Potential therapeutic target	[44]
	Cervical Cancer	Xq12 amplification causes overexpression; high expression inhibits proliferation (context-dependent)	E2F-KIF4A, KIF4A-KIF11/PRC1	High expression correlates with better OS/PFS; low expression linked to high CNV burden, NOTCH1 mutations	High expression inhibits proliferation; low expression promotes genomic instability	Prognostic marker; low expressers may benefit from immunotherapy	[106, 107, 111]
	Cholangiocarcinoma (CCA)	Modulates activated memory T cells and mast cell infiltration	Immune infiltration pathways	Overexpression, associated with poor survival	Promotes immune tolerance	Prognostic marker; potential guide for immunotherapy	[102]
	Oral Squamous Cell Carcinoma (OSCC)	Upregulates CCL2 to recruit M2 macrophages; knockdown activates SAC	KIF4A-CCL2/CCR2, SAC pathway	Overexpression, correlates with tumour size	Promotes proliferation, immune evasion; knockdown causes G2/M arrest	Potential therapeutic target	[103, 104]
	Pancreatic Ductal Adenocarcinoma (PDAC)	Involved in cell cycle; part of 36-gene prognostic signature	Cell cycle pathways	Overexpression, associated with shorter survival; 36-gene signature outperforms TNM staging	Promotes proliferation	Independent prognostic factor; aids risk stratification	[105]
	Pleural Mesothelioma (PM)	Regulates DNA replication; part of 3-gene model with UHRF1, NEK2	DNA replication pathways	Overexpression; 3-gene high-risk group associated with shorter survival	Promotes proliferation	Prognostic factor; part of 3-gene model	[112]
	Ewing Sarcoma (ES)	Regulates cell cycle; part of 7-gene prognostic signature	Cell cycle pathways	Overexpression, associated with poor prognosis	Promotes proliferation	Prognostic marker; potential target	[115]
Non-Cancer Diseases	Neurodevelopmental Disorders (XLID100)	Loss-of-function mutations disrupt neuronal transport, synaptic balance	Axonal transport, synaptic pathways	Missense/splice/frameshift mutations; Xq13.1 duplication in FIS patients	Causes intellectual disability, multi-organ abnormalities	Basis for XLID100 diagnosis	[12, 13, 121, 122]
	Neuropsychiatric Disorders (Epilepsy/ID)	Epilepsy: R728Q disrupts PARP1 pathway; ID: impairs synaptic balance	PARP1-TrkB-KCC2, synaptic pathways	Epilepsy: R728Q mutation; ID: loss-of-function	Lowers seizure threshold; causes cognitive impairment in ID	Therapeutic target (KIF4A/PARP1); basis for genetic diagnosis	[121, 123]
	ANCA-Associated Vasculitis (AAV)	Anti-KIF4A antibodies involved in autoimmunity	Autoimmune pathways	High anti-KIF4A antibody positivity in patients	Promotes autoimmune response	Potential serological diagnostic marker	[124]
	Psoriasis + Crohn’s Disease (comorbidity)	Shared hub gene; etoposide downregulates its expression	Cell cycle, immune pathways	Shared overexpression; correlates with disease activity	Promotes comorbidity progression	Comorbidity mechanism target; etoposide as reference	[125]
	Idiopathic Pulmonary Fibrosis (IPF)	Promotes fibrosis; Jinshui Huanxian Formula (JHF) downregulates its expression	Fibrosis pathways	Overexpression in fibrosis models; downregulated after JHF treatment	Promotes fibrosis; JHF ameliorates pathology	Disease key gene; JHF target	[126]
	Dental Disease (X-linked anomalies)	Missense mutations affect cell migration, disrupt tooth development	Cell migration pathways	Missense mutations; expressed during tooth development	Causes taurodontism, microdontia, dens invaginatus	Basis for genetic diagnosis	[120]
	Reproductive Developmental Disorders	(1) Mutations cause sperm/embryo abnormalities; (2) Downregulated in IUA, inhibiting endometrial regeneration	Spermatogenesis, embryonic mitosis pathways	Loss-of-function mutations; downregulated in IUA lesions	Causes infertility, embryonic arrest; IUA inhibits endometrial regeneration	Basis for male infertility diagnosis; IUA mechanism target	[130–132]
	Viral Hepatitis (HBV/HDV)	Interacts with NTCP to promote viral infection	KIF4A-NTCP-HBV/HDV	Normally expressed in host; downregulated by RXR agonists	Enhances viral infection efficiency	Antiviral target; bexarotene as reference	[127]
	Connective Tissue Disease (cEDS)	Aberrant expression in fibroblasts, potentially involved in ECM remodelling	ECM remodelling pathways	Aberrant expression in cEDS patient fibroblasts	May exacerbate connective tissue abnormalities	Clue to pathological mechanism	[129]
	Type 2 Diabetes + Cancers (comorbidity)	Shared hub gene, links metabolism and tumour via p53/MAPK	p53, MAPK pathways	Overexpression in T2D and associated cancers	Promotes metabolism-associated tumour progression	Combination intervention target; comorbidity node	[133]
	COVID-19 + Digestive Cancers (comorbidity)	Differentially expressed, enriched in cell division pathways	Cell division pathways	Differentially expressed in COVID-19 and liver/colorectal cancers	May promote infection-associated tumour progression	Target for infection-tumour mechanism	[134]

## KIF4A as a target for clinical diagnosis and therapy

Building on the mechanistic and pan-disease insights discussed above, we next summarize the clinical implications of KIF4A, focusing on its applications as a biomarker and therapeutic target.

### Applications as a biomarker

#### Prognostic prediction

KIF4A itself is an excellent independent prognostic marker. Monitoring its mRNA or protein levels can facilitate patient risk stratification, enabling healthcare providers to identify individuals at high risk for recurrence, metastasis, and reduced survival rates. This information can guide the implementation of more aggressive adjuvant therapy strategies [6, 39, 46, 68, 72, 78, 135].

#### Diagnostic assistance

The significant differential expression of KIF4A between cancerous and normal tissues positions it as a promising diagnostic marker. Notably, multi-gene predictive models built around KIF4A and other key genes (e.g., UBE2C, TOP2A, CDCA3, CDCA5, ASPM) demonstrate extremely high diagnostic value. For example, in oesophageal cancer (ESCA), the combination of KIF4A, RAD51AP1, and CDKN3 serves as a robust marker, achieving an exceptional ability to differentiate tumour from normal tissue (AUC = 0.979)^84^. InccRCC, a 4-gene model comprising KIF4A, UBE2C, OTX1, and PPP2R2C showed great potential to distinguish tumour tissues from normal ones [119]. In HCC, a four-gene combination involving KIF4A, UBE2T, CDCA3, and CDCA5 can track disease progression from chronic active hepatitis B (CAH-B) and liver cirrhosis (LC) to HCC [117, 136]. Similar models have been validated in various cancers, including liver [117, 119, 136], kidney [98, 119, 137], and prostate cancer [46, 66, 67].

#### Treatment response prediction

Elevated levels of KIF4A may indicate treatment response, with high expression correlating with primary or secondary resistance to platinum-based chemotherapy [5, 10, 93] and endocrine [5]. Additionally, KIF4A’s connection to an immunosuppressive microenvironment could suggest a poor response to immune checkpoint inhibitors [9, 114]. In triple-negative breast cancer, high KIF4A expression is linked to increased sensitivity to PLK1 inhibitors, such as GSK461364^138^. In clear cell renal cell carcinoma (KIRC), a prognostic model based on KIF4A and related genes (UBE2C, OTX1, PPP2R2C) has shown that the risk score not only predicts patient outcomes but also positively correlates with the expression of immune checkpoints (e.g., PD-1, CTLA-4) and potential benefits from immunotherapy, indicating its utility in guiding treatment decisions for KIRC patients [119]. Conversely, lower expression of KIF4A or specific mutations may suggest that patients could benefit from certain treatments; for instance, cervical cancer patients with reduced KIF4A expression demonstrated better responses to immunotherapy [111]. Furthermore, KIF4A expression levels are significantly associated with sensitivity to various chemotherapeutic agents. Prediction models based on KIF4A expression can reliably predict tumour cell sensitivity to platinum-based agents, taxanes, and topoisomerase inhibitors, achieving an area under the curve (AUC) exceeding 0.85, thus providing a valuable tool for personalized chemotherapy regimens [138].

### Applications as a therapeutic target

Given its robust association with patient outcomes and treatment response, KIF4A’s utility extends beyond biomarker applications. Increasing experimental evidence suggests that direct or indirect targeting of KIF4A may provide meaningful therapeutic benefits.

#### Direct targeting strategies

Small Molecule Inhibitors: This field is in its early stages but has witnessed promising advancements. Database screenings and experimental validations have identified the small molecule compound WZ-3146 as an effective inhibitor of KIF4A function. In glioma models, both WZ-3146 treatment and KIF4A knockdown significantly inhibited tumour cell proliferation and migration while inducing apoptosis via Caspase-3 activation [8]. Moreover, researchers have successfully created a chimeric KIF4A protein that can be inhibited by the specific small molecule STLC by transplanting the allosteric inhibition site of Eg5 (Kinesin-5) into KIF4A’s motor domain. While this serves as a proof-of-concept tool, it lays the groundwork for future development of specific KIF4A inhibitors [25, 139].

Gene Silencing Therapy: Silencing KIF4A expression through siRNA, shRNA, or CRISPR-Cas9 technology (in vitro *and* in vivo mouse xenograft models) has been demonstrated to effectively inhibit tumour growth, reverse chemotherapy resistance, and enhance the immune microenvironment [4, 5, 9, 51, 90, 93]. This establishes a strong preclinical foundation for developing RNA interference (RNAi)-based therapies.

Targeting circKIF4A: The oncogenic circKIF4A, which is highly expressed in breast cancer (sponging miR-335 [31]) and glioma (sponging miR-335-5p [30]), can be targeted using antisense oligonucleotides (ASOs) or small molecules designed for degradation. This approach relieves circKIF4A’s sponge effect on specific miRNAs, indirectly inhibiting KIF4A expression and function [30, 31].

Exosome Delivery Therapy: Exosomes secreted by normal human chondrocytes can act as natural delivery vehicles, transporting miR-195 to osteosarcoma cells. miR-195 inhibits KIF4A expression by directly targeting its 3’UTR, leading to a reduction in tumour volume by over 40% in vivo models while significantly promoting apoptosis [90]. This presents a novel avenue for vesicle-based targeted therapy.

#### Comparison of exosome-based delivery with alternative targeting strategies for minimizing KIF4A-related toxicity

While exosome-based delivery systems offer a promising approach to targeting KIF4A with reduced systemic toxicity, particularly neurotoxicity resulting from its essential role in axonal transport and mitotic processes, the clinical translation of exosome therapeutics remains challenging. Exosomes possess several intrinsic advantages: their naturally low immunogenicity, biocompatibility, and inherent ability to cross biological barriers—including the blood–brain barrier—make them particularly attractive for delivering siRNAs or miRNAs directed against KIF4A [30, 31]. For example, exosomal delivery of miR-195 has demonstrated significant therapeutic efficacy in osteosarcoma, achieving over 40% tumor volume reduction in vivo. These findings underscore the potential of exosomes as precise and minimally invasive delivery vehicles capable of mitigating the systemic safety concerns associated with direct KIF4A inhibition.However, several major limitations currently impede the widespread clinical application of exosome therapeutics. Large-scale production and purification of exosomes remain technically difficult, with batch-to-batch variability affecting consistency and safety. Efficient loading of nucleic acids or small molecules into exosomes is challenging, and current techniques (electroporation, sonication, membrane permeabilization) often result in suboptimal loading efficiency or structural damage. Furthermore, exosomes lack intrinsic high-affinity targeting specificity; engineering surface ligands or modifying donor cells is usually required, adding complexity and regulatory burdens. Standardized quality-control metrics, validated manufacturing pipelines, and long-term stability data are still lacking, collectively limiting their readiness for clinical translation [31].In comparison, antibody–drug conjugates (ADCs) offer much higher target specificity through monoclonal antibody recognition of tumor-specific antigens, enabling localized delivery of cytotoxic payloads. ADCs bypass many of the manufacturing constraints encountered with exosomes and have clearer regulatory pathways, with several approved for clinical use. However, ADCs carry intrinsic risks of on-target/off-tumor toxicity, limited payload capacity, premature linker cleavage, and high production costs. Moreover, ADCs rely on the presence of surface antigens, which may not be uniformly expressed across tumors where KIF4A is intracellular and nuclear.Localized delivery methods, such as intratumoral injection of siRNA nanoparticles, polymer hydrogels, or viral vectors, offer the safest systemic profile. These approaches minimize off-target effects and reduce neurotoxicity risk, since KIF4A inhibition is spatially restricted [31]. Nonetheless, localized delivery is feasible only for accessible or early-stage tumors and is impractical for metastatic or deeply seated cancers—particularly those such as gliomas or disseminated gastrointestinal tumors, where KIF4A is highly oncogenic.

#### Indirect targeting and combination therapy strategies

Targeting Upstream Regulators: Given that KIF4A transcription is driven by factors such as FOXM1 and YAP, employing YAP inhibitors (e.g., verteporfin) [24] or FOXM1 inhibitors represents a viable strategy for indirectly downregulating KIF4A expression.

Disrupting Oncogenic Axes: Precision medicine can benefit from developing inhibitors that target key protein-protein interaction interfaces. For instance, creating small molecules or peptides that disrupt KIF4A-AR or KIF4A-TPX2 interactions could effectively treat conditions like CRPC [5] or endometrial cancer [4].

Combination Therapy: This strategy holds significant near-term clinical potential. Combining KIF4A silencing or inhibition with standard therapies can produce synergistic effects. For example, KIF4A inhibitors used alongside cisplatin [93], oxaliplatin [43], 5-FU [10], or enzalutamide [5] may help overcome resistance. Additionally, aspirin has been shown to inhibit ESCC progression by downregulating the ATAD2/KIF4A axis [83]; while etoposide ameliorates psoriasis and Crohn’s comorbidity by regulating KIF4A [125]. Importantly, as KIF4A inhibition can enhance the immune microenvironment (e.g., reducing MDSC infiltration), combining it with immune checkpoint inhibitors (e.g., anti-PD-1/PD-L1 antibodies) could represent an effective strategy to convert “cold” tumours into “hot” ones, thereby overcoming immunotherapy resistance [9].

#### Other intervention strategies

miRNA Mimics: Developing nanomedicines that deliver miRNA mimics (e.g., miR-335, miR-379-5p) to restore their natural inhibition of KIF4A has the potential to reduce tumour growth by 35%-50% in models such as breast cancer. This represents another promising therapeutic approach [27, 28].

Exosome-Based Therapy: As previously mentioned, utilizing normal chondrocyte-derived exosomes to deliver miR-195 effectively targets KIF4A in osteosarcoma cells, leading to inhibited growth and enhanced apoptosis [90].

Neoantigen-Based Immunotherapy Strategy: Integrated multi-omics and AI analyses have identified KIF4A as one of the most critical immunogenic neoantigens in CRC. Personalized vaccines designed based on this finding could significantly enhance survival rates for patients exhibiting high immune gene expression subtypes [79].

### Innovativeness and prospects of KIF4A as a therapeutic target

Although these therapeutic strategies show significant promise, several challenges remain before KIF4A can be safely and effectively translated into clinical interventions. These considerations highlight key opportunities and obstacles that will shape future research and development.KIF4A is recognized as a promising therapeutic target due to several distinctive features. Its functional duality and context-dependency create avenues for precision therapy; it acts as both an “oncogene” and an “essential gene.” This duality, while complex, allows for the potential targeting of tumours specifically. Cancer cells often demonstrate a greater reliance on KIF4A for proliferation compared to normal cells, presenting a theoretical therapeutic window. Interestingly, KIF4A’s role can shift in certain contexts, such as cervical cancer [111], highlighting the need for the development of biomarker-based strategies to tailor precision medicine approaches. Additionally, KIF4A has notable pan-cancer traits, being overexpressed and linked to poor prognosis in numerous malignancies like endometrial [4, 63], prostate [5, 65], glioma [7, 8], hepatocellular [71, 74], colorectal [10, 43], breast [27, 31], and lung cancers [38, 93], meaning targeting strategies could benefit a wide range of cancer patients. Notably, KIF4A also plays a crucial role in modulating the immune microenvironment. By upregulating CXCL5 expression, KIF4A recruits MDSCs which dampens anti-tumour immunity [9]. Consequently, targeting KIF4A may not only inhibit tumour growth directly but also help reverse immunosuppression, transforming “cold” tumours into “hot” ones. This makes the combination of KIF4A targeting with immune checkpoint inhibitors an appealing strategy.

Despite its potential as a therapeutic target, clinical translation of KIF4A is still nascent, with no drugs specifically targeting it currently undergoing clinical trials. Ongoing research is dedicated to identifying novel inhibitors and combination therapies. Small molecules such as WZ-3146 have shown anti-proliferative and pro-apoptotic effects in glioblastoma models, but their mechanisms, pharmacokinetics, and safety profiles require thorough investigation [8]. The strategy of chemically modifying KIF4A to enhance inhibitor sensitivity is currently limited to research applications [25, 139]. More focus is being directed toward indirect targeting and drug combinations, such as improving enzalutamide efficacy in CRPC by inhibiting KIF4A [5], or overcoming chemoresistance in CRC with oxaliplatin and 5-FU^10,43^. A significant hurdle for KIF4A’s clinical translation is its dual biological roles—functioning as an “oncogene” in cancer yet serving as an “essential gene” for normal cellular processes such as mitosis and nervous system function [3, 12, 22]. Systemic inhibition could result in adverse effects, including mitotic disruption and neurotoxicity. Therefore, developing targeted delivery systems, such as exosome-based siRNA or miRNA delivery [90], is essential to advance its clinical application.

To further clarify KIF4A’s potential as a diagnostic and therapeutic target, we have compiled various strategies, mechanisms, and current evidence supporting its use, emphasizing its potential to enhance patient outcomes (Table 2).

**Table 2:** Summary of the clinical utility of KIF4A in diagnosis and therapy

Application Direction	Strategy Type	Specific Method/Target	Core Mechanism/Advantage	Current Evidence Level	Major Challenges	Ref.
Biomarker	Prognostic Prediction	KIF4A mRNA/protein expression	Independent prognostic factor, significantly associated with recurrence, metastasis, survival	Validated across multiple cancers	Lack of standardized detection protocols, limited comparability	[6, 39, 46, 68]
	Diagnostic Assistance	KIF4A + UBE2C/TOP2A/CDCA3 etc. combinations	Significant difference between cancer and normal tissue; multi-gene models enhance efficacy (e.g., ESCA AUC = 0.979)	Bioinformatics + partial clinical validation	Insufficient prospective validation, not yet routine in clinical diagnostics	[84, 117, 119]
	Efficacy Prediction	KIF4A high expression	Predicts chemotherapy/endocrine therapy resistance, poor response to immunotherapy	Retrospective studies	Requires multicentre prospective validation, lacks dynamic monitoring schemes	[5, 9, 10, 126]
Therapeutic Target	Direct Targeting	Small molecule inhibitor (WZ-3146)	Inhibits KIF4A function, induces apoptosis, suppresses proliferation	Preclinical (cell + animal models)	Selectivity and toxicity need optimization	[8]
		Gene silencing (siRNA/shRNA/CRISPR)	Inhibits tumour growth, reverses chemotherapy resistance, improves immune microenvironment (reduces MDSCs)	Preclinical validation in multiple cancers	Delivery systems need optimization, in vivo safety requires verification	[4, 5, 9, 90]
		Targeting circKIF4A (ASO/small molecule)	Degrades circKIF4A, relieves miRNA sponge effect, indirectly suppresses KIF4A	Preclinical (breast cancer)	In vivo stability of oligonucleotide drugs	[30, 31]
		Exosomal delivery (chondrocyte exosomes delivering miR-195)	Natural low-immunogenicity carrier, reduces osteosarcoma volume by 40%+	Preclinical (osteosarcoma animal model)	Difficulties in large-scale production, loading efficiency needs improvement	[90]
	Indirect Targeting	Upstream regulation (YAP/FOXM1 inhibitors)	Indirectly downregulates KIF4A expression, utilizing existing inhibitors	Preclinical	May trigger compensatory mechanisms	[24, 74]
		Disrupting protein interactions (KIF4A-AR/KIF4A-TPX2)	Blocks oncogenic axis, reverses CRPC resistance	Preclinical (cell + partial animal experiments)	Difficulty in developing protein-protein interaction inhibitors, insufficient specificity	[4, 5]
	Combination Therapy	Combined with chemotherapy (cisplatin/oxaliplatin/5-FU)	Synergistic effect, reverses resistance	Preclinical	Optimal combination and dosing sequence need exploration	[10, 43, 93]
		Combined with immunotherapy	KIF4A inhibition improves immune microenvironment (reduces MDSCs), turns “cold” tumours “hot”	Preclinical (bladder cancer)	Risk of immune-related adverse events	[9]
	Other Strategies	miRNA mimics (miR-335/miR-379-5p)	Restores miRNA suppression, inhibits breast cancer growth by 35%–50%	Preclinical (breast cancer models)	In vivo delivery efficiency and stability	[27, 28]
		Exosomal delivery (e.g., miR-195)	Natural delivery system, high targeting specificity, low immunogenicity	Preclinical (osteosarcoma)	Large-scale production, standardization, drug loading efficiency	[90]
		Neoantigen-based immunotherapy	Personalized vaccines may improve survival in immune gene-high subtypes	Supported by multi-omics and AI analysis	Challenges in personalized vaccine production, limited applicable population	[79]
Innovation and Prospect	-	Context-dependent target	May act as tumour suppressor in cervical cancer, requires biomarker-based stratification	Clinical observation	Mechanism unclear, requires cautious application	[111]
	-	Pan-cancer target	Overexpressed in multiple cancers, potential for broad applicability	Supported by multi-omics data	Risk of tissue-specific toxicity	[4, 5, 8, 9, 71]
	-	Immune microenvironment modulation	Links tumour cell proliferation and immune suppression, high potential for combination immunotherapy	Mechanistically elucidated	Complex roles of immune cell subsets require further exploration	[9]

## Summary and outlook

Collectively, the mechanistic, pathological, and translational evidence discussed above positions KIF4A as a compelling target across human diseases. The following section synthesizes these insights and outlines future directions needed to bridge current knowledge gaps and advance KIF4A-based precision medicine.KIF4A has emerged as a critical molecule from fundamental cell biology research, demonstrating significant clinical translational potential. It exemplifies the intricate connection between basic cellular processes and human disease pathology. This review systematically outlines the biological characteristics of KIF4A, summarizes its central roles in neoplastic diseases as an oncogene, and explores its involvement in neurodevelopmental disorders. For the first time, it integrates KIF4A’s functions in non-neoplastic conditions, moving beyond a cancer-centric perspective while thoroughly discussing its clinical translation prospects.

The future application of KIF4A appears promising. KIF4A-based multi-gene signatures, such as KIF4A + RAD51AP1 + CDKN3 in oesophageal cancer, are poised to enter clinical practice as routine tools for molecular diagnostics and prognostic predictions, enabling more precise patient stratification. Moreover, therapeutic strategies that target KIF4A, especially combination therapies involving chemotherapy, targeted therapy, or immunotherapy, provide exciting new directions to address two significant challenges in cancer treatment: therapy resistance and immune evasion.

Nonetheless, several challenges persist. The primary concern is its functional duality and context-dependency. While KIF4A is typically detrimental in most cancers, it may play a protective role in specific contexts, such as cervical cancer [111], necessitating careful consideration of cancer types and molecular backgrounds when developing targeting strategies. Additionally, KIF4A’s essential roles in normal mitosis and nervous system function imply that systemic inhibition could lead to on-target side effects, including cytotoxicity and neurotoxicity. Thus, developing targeted delivery systems (e.g., exosome-based siRNA/miRNA delivery [90]) is crucial for advancing its clinical application. Furthermore, most of the existing research remains at the preclinical stage, underscoring the urgent need for more translational research to convert robust laboratory findings into candidate drugs and clinical trials.

Future studies should focus on elucidating the mechanisms behind KIF4A’s functional switching in various microenvironments and accelerating the development of highly selective tumour delivery systems to enhance treatment safety [90]. From a translational perspective, utilizing KIF4A expression profiles to develop diagnostic and prognostic models has demonstrated good clinical applicability, and its characteristic pan-cancer upregulation is particularly advantageous for formulating broad-spectrum targeting strategies [4, 5, 9]. strategies. Additionally, regulating the immune microenvironment presents a promising avenue for KIF4A-targeted therapies. Current evidence suggests that inhibiting KIF4A can reduce MDSC recruitment and enhance cytotoxic T cell infiltration [9], providing a rationale for combining it with immune checkpoint inhibitors to overcome barriers in tumour immunotherapy resistance. Recent single-molecule techniques have revealed that KIF4A can influence the run length and speed of other motor proteins at the micrometre scale by altering the microtubule lattice state [37]. This “remote communication” mechanism, which operates independently of direct protein interactions, represents a novel paradigm for intracellular regulation and offers a revolutionary approach for future therapies focused on physical rather than chemical modulation [37].

In conclusion, KIF4A play crucial roles in diverse diseases, and is increasingly being studied. As our understanding of its functional mechanisms deepens and innovative treatment modalities continue to emerge, targeting KIF4A holds the potential to offer new hope to many patients, particularly those with cancer facing treatment challenges. The research progress on the roles of KIF4A—from a fundamental mitotic regulator to a multifunctional hub in disease—illustrates the principles of translational medicine. In future, it holds great potential for developing new strategies and tools for precision medicine.

## Data Availability

This manuscript is a review article and does not contain any original datasets. All data and findings discussed herein are based on previously published studies, which are fully referenced. Readers can access the underlying data by consulting the referenced publications.
